# Physiology and the Olympics

**DOI:** 10.1113/EP092580

**Published:** 2025-10-15

**Authors:** Toby Mündel

**Affiliations:** ^1^ Department of Kinesiology Brock University St. Catharines Ontario Canada

## 1 INTRODUCTION

Physiology plays a key role in understanding and optimizing human physical performance. For many athletes, the Olympic Games remain the pinnacle of achievement. Beyond public interest and return on (state) investment, studying the physiology of athletes provides a unique insight into the capabilities of the various systems (e.g., muscular, cardio‐ and cerebrovascular, respiratory) that can support basic and clinical research. This was the premise upon which a call was made for the current Special Issue on ‘Physiology and the Olympics’, focused on advancing our physiological understanding of the science behind preparation for, competition in, and recovery from human performance. This call was answered comprehensively: (1) 18 papers in total, whose corresponding authors span nine countries (Belgium, Canada, France, Germany, Greece, Italy, Poland, UK and USA); (2) article categories including Myths and Methodologies (1), Short Communication (1), Case Reports (3), Reviews (4) and Research Articles (8); (3) subject areas that embrace Cardiovascular Control, Endocrinology and Metabolism, Environmental and Exercise, Muscle and Neuroscience; and (4) athlete (participant) categories that comprise cycling, running, jumping, throwing, swimming, football (soccer), sailing, alpine skiing, kayaking, weight‐ and power‐lifting, CrossFit, esports, speed‐skating, skeleton, biathlon, triathlon and decathlon. No matter your interest, this Special Issue has something to whet your physiological appetite!

## 2 AEROBIC CAPACITY AND PREDICTION OF PERFORMANCE

A case report by Zubac et al. (2025) provided a retrospective analysis of world‐class Laser Standard sailors through cardiopulmonary exercise testing. These sailors are renowned for their agile ‘hiking’ skills, whereby quasi‐isometric bilateral and multi‐joint movements account for up to 90% of the upwind time, strongly relate to neuromuscular fatigue and play a pivotal role in sailing performance. They report that well‐balanced aerobic power is vital for superior sailing performance, whilst oxygen pulse measures over 6 years were noted not to change, indicative of compensatory cardiovascular mechanisms.

Bouten et al. (2025) review the literature on apnoea as a novel training method to improve aerobic performance, with only long and intense apnoea protocols driving haematological adaptation (i.e., elevation in erythropoietin, reticulocytes) in experienced breath‐holders, and with restricted breathing during exercise potentially proving a low‐cost alternative training method for elite (world‐class) athletes.

In their review, Paris et al. (2025) ask whether being born at high altitude creates an ergogenic advantage for sea‐level endurance performance. The authors distinguish between: (1) the Indigenous highlander (e.g., Andean mountain or Tibet plateau natives); (2) the highland newcomer (Han migrants to Tibet or those from Leadville, CO, USA); and (3) the highland sojourner. Focusing on pulmonary physiology from the perinatal period onwards, Paris et al. (2025) conclude that genomic and developmental adjustments associated with highland birth might present sea‐level endurance benefits if altitude‐related maladaptation is avoided.

Staying with environmental adaptation, Tyler and Notley (2024) highlight key methodological considerations required when interpreting the time course of complete heat adaptation, whilst also offering an improved means to induce and quantify the magnitude of adaptation.

An emerging component of endurance performance is durability, defined as the ability to preserve physiological function during prolonged exercise. Hunter et al. (2025) review the current approaches to measure durability, which include assessing physiological characteristics at rest or following prolonged exercise, and quantification of the internal‐to‐external work rate ratio decoupling during exercise. The authors encourage greater protocol rigour and consistency in approach that should result in better physiological understanding and interventions that might enhance it for researchers and practitioners in this emerging field.

Respiratory muscle strength and endurance are known to be important components of athletic performance, and Kowalski et al. (2025) present the first comprehensive reference values for the S‐Index Test, an evaluation of respiratory muscle function based on maximum dynamic inspiratory pressure, in well‐trained athletes, e‐sport athletes and age‐matched control subjects (*n *= 597). Higher S‐Index Test results were observed in males than in females, in well‐trained athletes than in age‐matched control subjects, and within well‐trained athletes, age and training status were positively correlated with test results.

Battles et al. (2025) ask the question: when selecting and developing elite decathletes, which are the most important event strengths to track? Using publicly available data (World Athletics) between 2001 and 2022, the authors use gamma generalized linear regression models, the first‐season best mark and the improvement in each event related to all‐time career best. The most crucial event for identifying the future potential of athletes is the pole vault, followed by javelin, long jump and shot put. For optimal career development, athletes obtain the most return by focusing on pole vault and long jump improvement.

## 3 STRENGTH AND HIGH‐INTENSITY EXERCISE: BIOLOGICAL SEX

Moving on from aerobic performance, Kazan et al. (2025) ask the question: which insertion and deletion (indel) variants are functional and related to power athlete status? This first exome‐wide association study found that the D allele of the *MDM4* gene rs35493922 polymorphism was over‐represented in two independent cohorts of power athletes in comparison to endurance athletes and control subjects and positively associated with fat‐free mass and cross‐sectional area of fast‐twitch muscle fibres. Thus, *MDM4* is thought to be a potential regulator of muscle fibre size and specification.

Lee et al. (2025b) provide a double‐blind case report of the metabolic responses to ingestion of hydrolysed collagen prior to resistance exercise in a young, female CrossFit athlete across two consecutive menstrual cycles when oestrogen was low (onset of menses) and high (late follicular phase). Their results revealed that when circulating oestrogen was high, whole‐body collagen synthesis was low(er), and that hydrolysed collagen ingestion offset this physiological disadvantage when oestrogen was high and was even more advantageous when oestrogen was low. These results provide evidence for the potential of hydrolysed collagen to protect connective tissue from injury in naturally menstruating strength athletes. Building on these results, the same group investigated whether 10 weeks of pre‐season football (soccer) training could confer greater changes in patellar tendon mechanical and material properties with hydrolysed collagen than an energy‐matched placebo in professional female athletes (Lee et al., 2025a). The authors observed that collagen supplementation resulted in greater increases in tendon stiffness and Young's modulus, further strengthening their case for improving athletic performance and mitigating injury risk in athletes during high‐intensity exercise.

Staying with female football (soccer) players, McHaffie et al. (2025) aimed to determine whether the prevalence of low energy availability in this cohort is overestimated owing to inaccuracies through self‐reporting of dietary intakes when compared with quantifying energy intake and availability using the doubly labelled water method. During a 9–10 day ‘training camp’ representing their national team, data from 45 adolescent football players showed the potential to underestimate energy intake significantly when using the self‐report method, perhaps misrepresenting and overestimating the prevalence of low energy availability, an underlying cause of relative energy deficiency in sport.

The final report concerning female athletes comes from Collantes et al. (2025), who set out to evaluate the improvements in male and female world records in four running events (100, 200, 400 and 800 m) and two throwing events (discus and shot‐put) over time, and the influence of doping programmes on these records from an online database (worldathletics.org). The authors conclude that androgenic–anabolic steroids are likely to have played a substantial role in world‐best athletics performances, especially for females, based on three main findings: (1) the performance year associated with top athletes occurred earlier for females than for males, especially for the 800 m and shot‐put; (2) for females, world records did not progress after 1990, when random out‐of‐competition and systematic in‐competition drug testing began; and (3) there was a greater representation of countries with known state‐sponsored doping programmes, especially among females.

## 4 MUSCLE, SLEEP AND SLALOM SKIING

During high‐intensity exercise, there exists a hyperbolic relationship between time to exhaustion and power output, which can be described (mathematically) by a power asymptote, critical power, and a curvature constant (*W′*), with critical power demarcating the heavy/severe domains of exercise intensity (Poole et al., 2016). This concept and these parameters can be of use to athletes, coaches and physiologists with an interest in fatigue development and causal mechanisms. However, whether and how these parameters relate to skeletal muscle mitochondrial and respiratory function has not been forthcoming and was the aim of investigation by Peden et al. (2025). Participants performed three to five constant‐load tests to failure to identify critical power and *W′*, and these were correlated with vastus lateralis citrate synthase activity and mitochondrial complexes I–IV. Citrate synthase activity was positively correlated with critical power but not with *W′*, suggesting that critical power was more likely to be determined by mitochondrial content than respiration, whereas some components of mitochondrial respiration were positively correlated with *W′*. Thus, skeletal muscle mitochondrial oxidative capacity is related to critical power, strongly determined by mitochondrial content.

Villanova et al. (2025) determined whether trained swimmers have greater triceps brachii oxidative capacity and sensitivity to reduced O_2_ availability than recreational swimmers. The authors compared recreational/trained, national and international/world‐class swimmers for their 100 m freestyle performance and observed their triceps brachii characteristics through intermittent occlusions in well‐oxygenated and low‐O_2_ availability conditions using near‐infrared spectroscopy. They found that international/world‐class swimmers had higher oxidative capacity than recreational swimmers and that estimates of muscle oxidative capacity were positively correlated with training volume and swimming performance, although sensitivity to reduced O_2_ availability was not different between the groups. These results suggest that the high triceps brachii oxidative capacity in international/world‐class swimmers is supported by a proportional adaptation in capillary network and O_2_ delivery that argues against an O_2_ diffusion limitation.

Another study on elite swimmers, by Arc‐Chagnaud et al. (2025), investigated whether partial body cryostimulation affected sleep and recovery parameters during an intense training period. During a 2 week period, swimmers were randomly assigned to receive 3 min of head‐out cryo‐cabin exposure (−110°C) after their evening training session for 5 days consecutively (from Monday to Friday), whilst during the other week they did not receive any intervention (control). The authors report that cryostimulation reduced perceived anxiety, tiredness and depression, and that increased slow‐wave sleep was also noted during the first sleep cycle by use of a validated headband (Dreem 2), although other subjective and objective measures of sleep remained unaffected. Saliva concentrations of C‐reactive protein were also lower, and when the authors split the group further into ‘better’ and ‘worse’ sleep according to the median division of time spent awake during the night, they noted that the poorer sleepers obtained a benefit to their sleep following cryotherapy.

Botonis et al. (2025) provide us with a critical examination of the current knowledge base concerning rapid transmeridian air travel on physical performance, time line of recovery and dissipation of jet lag symptoms. They conclude that aspects of anaerobic performance and strength are negatively affected by long‐haul transmeridian travel, whereas evidence on endurance exercise remains equivocal; negative effects are more pronounced after eastbound travel, and recovery of performance may take >3 days. However, many factors influence these effects that have not been elucidated fully, such as real versus simulated travel, the number of time zones crossed and whether during normal wake time or sleep, whether performance is assessed or factors related to performance, and whether this testing takes place at destination time or ‘body time’.

Varesco et al. (2025) report an assessment of the impact of long‐haul travel across 13 time zones (from Montréal to Asia for World Cup races) by the Canadian Short‐Track Speed Skating Team on sleep patterns, rest–activity circadian rhythms, neuromuscular function and race performance. Actigraphy data showed that in comparison to baseline, athletes slept less during travel and competition days and more during their stay in Asia. Sleep efficiency and countermovement jump performance were greater in Asia than at baseline, rest–activity circadian rhythms shifted from late evening to mid‐afternoon, and race performance was unaffected. These results demonstrate that despite possible sleep debt accrued through long‐haul travel, athletes recover by the fifth day without impacting their performance and highlight their ability to manage jet lag.

Alhammoud et al. (2025) examine Olympic medallist slalom skiers during repeated slalom training runs with continuous recording of right knee and hip angles, turn and run times. Joint kinematics showed minor alterations across eight slalom runs, and in the absence of changes in performance (turn and run times), are interpreted as adaptations in style rather than less functional technique in fatigued conditions.

## 5 SUMMARY

This Special Issue displays the depth and breadth of physiological research into the performance of athletes from around the globe, some of which will have immediate and direct impact on preparation for the next Olympic Games, beginning in February 2026 in Northern Italy. It remains for me to thank all the authors, reviewers and Journal/Society staff who have made this Special Issue possible, and I hope that you enjoy reading its contents.

## AUTHOR CONTRIBUTIONS

The author conceived, wrote and approved all versions of the text, and agrees to be accountable for all aspects of the work.

## CONFLICT OF INTEREST

The author declares that he has co‐authored an article that appears in the Special Issue. This article was handled and reviewed independently.

## FUNDING INFORMATION

The author is supported by the Canada Research Chairs Program.
